# Smartphone Dependence and Body Dissatisfaction in Adolescents

**DOI:** 10.1192/j.eurpsy.2026.10838

**Published:** 2026-07-31

**Authors:** T. Bernardo, C. Bento, A. Macedo, A. T. Pereira

**Affiliations:** 1 University Clinic of Paediatrics; 2 Institute of Psychological Medicine, Faculty of Medicine University of Coimbra, Coimbra, Portugal

## Abstract

**Introduction:**

Adolescence is a period characterized by several physical and psychological changes that influence body image. Body dissatisfaction appears to be associated with excessive smartphone use, as it promotes social comparison and search for others’ validation. Moreover, smartphone dependence has several characteristics that are like other addictions, being adolescents with low self-esteem and particularly vulnerable to its dependence.

**Objectives:**

The present work aimed to understand the relationship between smartphone dependence, body dissatisfaction, and self-esteem in adolescents from Coimbra.

**Methods:**

259 adolescents (59.85% girls), aged 12.93±1.161, answered some demographic questions and the Portuguese versions of the Mobile Phone Dependence Test, the Contour Drawing Rating Scale, and of the Rosemberg Self Esteem Scale. A cross-sectional, observational and correlational study was conducted in two schools of Coimbra. To analyse the different variables, a descriptive analysis, comparison tests and Pearson correlation were used.

**Results:**

47.70% of the girls and 29.80% of the boys wanted to have a small figure (wanted to be thinner). Body dissatisfaction was noticeably prevalent in our female sample [Boys - Real Image: 5.14 ±1.178 vs. Desired Image: 5.05 ± 0.805 t (103) = 0.928; p= 0.356] *vs.* [Girls - Real Image: 3.84 ±1.618 vs. Desired Image 3,26 ± 1,305 t (154) = 4.516; p< 0.001]. In girls, phone dependence was associated with lower self-esteem (r=-0.302, p<0.001); and higher body dissatisfaction (r=0.215, p=0.007). In boys, phone dependence was associated with lower self-esteem (r=-0.515, p<0.001). Furthermore, in both genders, self-esteem was negatively correlated with the phone dependence dimensions: abstinence (Boys: -0.432, p<0.001; Girls: -0.239, p=0.003), excessive use (Boys: -0.420, p<0.001; Girls: -0.296, p<0.001), and tolerance (Boys: -0.496, p<0.001; Girls: -0.284, p<0.001). In boys, self-esteem was negatively correlated with economic problems too (Boys: -0.328, p<0.001).

**Conclusions:**

The results emphasize the importance of understanding the risks associated with smartphone problematic use in adolescence, particularly its impact on body satisfaction and psychological health. In conclusion, intervention strategies focused on the literacy on adequate smartphone use, the improvement of self-esteem and body acceptance will be crucial to promote a healthier development of adolescents.

**Disclosure of Interest:**

None Declared

